# Three new endemic species of *Lepanthes* (Orchidaceae, Pleurothallidinae) from the highlands of Ecuador

**DOI:** 10.3897/phytokeys.180.62671

**Published:** 2021-08-09

**Authors:** Francisco Tobar Suarez, María Fernanda López, María José Gavilanes, Marco Federico Monteros, Tatiana Santander García, Catherine Helen Graham

**Affiliations:** 1 Área de Investigación y Monitoreo de Avifauna, Aves y Conservación – BirdLife en Ecuador, Quito, Ecuador Área de Investigación y Monitoreo de Avifauna Quito Ecuador; 2 Biodiversity and Conservation Biology Unit, Swiss Federal Research Institute WSL, Zurich, Switzerland Instituto Nacional de Biodiversidad, Herbario Nacional del Ecuador Quito Ecuador; 3 Instituto Nacional de Biodiversidad, Herbario Nacional del Ecuador QCNE, Quito, Ecuador Pontificia Universidad Católica del Ecuador Ibarra Ecuador; 4 Herbario HPUCESI, Pontificia Universidad Católica del Ecuador, Sede Ibarra, Ibarra, Ecuador Fundacion EcoMinga Baños Ecuador; 5 Fundacion EcoMinga, 270 12 de noviembre and Luis A Martínez, Baños, Tungurahua, Ecuador Swiss Federal Research Institute Zurich Switzerland

**Keywords:** El Oro, endemism, evergreen montane forest, Imbabura, *
Lepanthes caranqui
*, *
Lepanthes microprosartima
*, *
Lepanthes oro-lojaensis
*, páramo, Pichincha

## Abstract

Three new species of *Lepanthes* from Ecuador are described and illustrated. These additions to the Ecuadorean flora were recorded in evergreen montane forest and páramo as part of three different research projects conducted during the last five years (2016–2021). *Lepanthesoro-lojaensis* was discovered in the southwest of El Oro province and is similar to *L.jimburae*, differing mainly in the much smaller plants, inflorescences and floral parts. *Lepanthesmicroprosartima* from the western slopes of Pichincha volcano in northern Ecuador resembles *L.obandoi* but differs in the coloration of the leaves, the inflorescence that are shorter than the leaves and the smaller floral appendix. *Lepanthescaranqui*, found in eastern Pichincha and Imbabura, is most similar to *L.pachychila* but differs from it in its much larger plants and different shape of the petals and the floral appendix. Preliminary assessments of the conservation status of the three taxonomic novelties are provided, using the IUCN Red List Categories and Criteria.

## Introduction

Pleurothallidinae Lindl., with over 12,000 names published and around 5,100 currently accepted species, is the largest orchid subtribe worldwide (Karremans 2016). *Lepanthes* Sw. is one of the most diverse genera of the subtribe, and the estimated number range between 1,100 and 1,200 species (Larsen et al. 2018; Baquero et al. 2019; Bogarín et al. 2020), accounting for more than 20% of the species (Karremans 2016). *Lepanthes* is restricted to the Neotropics, ranging from the Antilles and southern Mexico through the Andes south to Bolivia, with a few species known from Brazil (Luer and Thoerle 2012; Larsen 2014). The largest number of species is concentrated in the Andes of Colombia, Ecuador and Peru (Damian and Larsen 2017; Bogarín et al. 2020), but an important number of species is also distributed in Costa Rica, Panama (Salazar and Soto-Arenas 1996; Bogarín et al. 2020). In Ecuador, this genus includes about 350 species, of which 240 are considered endemic to the country (Dodson and Luer 2011; Baquero et al. 2019). Nevertheless, their richness is far from being fully inventoried, as new species are continuously being discovered and described as the country’s forests continue to be explored (Thoerle and Hirtz 2015; Baquero 2018; Baquero et al. 2018; Tobar et al. 2018; Zambrano and Solano 2019).

Three new species: *Lepanthesmicroprosartima* Tobar & M.J.Gavil., *Lepanthescaranqui* Tobar & Monteros and *Lepanthesoro-lojaensis* Tobar & M.F.Lopez are described and illustrated here. These additions belong to the subgenus Lepanthes, sect. Lepanthes, which contains more than 243 spp. in Ecuador, and thus by far the largest in the genus (Luer and Thoerle 2012); *Lepanthes* subsections *Lepanthes* and *Breves* it subdivided into series, based largely on the morphology of the genus in Ecuador. An extensive examination of the genus beyond the borders of Ecuador demonstrated that these series were untenable, therefore (Luer 1993; Luer 1996; Luer 2010), we place the three species into subsection Lepanthes without further division.

These novelties were discovered and collected as part of three different research projects conducted during the last five years (2016–2021), including “The Ecology of Plant and Hummingbird Interactions Project (EPHI),” carried out in the western slopes of Pichincha province; “El Oro Biodiversity Project,” conducted in southwestern Ecuador; and the “Floristic Inventory of La Carboneria forest remnant,” in eastern Imbabura and Pichincha provinces. The discovery of these new species demonstrates the importance of continuing the botanical exploration of a mega-diverse and incompletely inventoried country such as Ecuador.

## Materials and methods

Plants were photographed in situ and subsequently pressed and dried, and deposited at QCA and QCNE (acronyms according to Thiers 2018 continuously updated). Photographs were taken using a Nikon D100 digital camera equipped with a 105 mm macro lens (Nikon). Morphological observations and measurements were made from live and alcohol-preserved material. The samples were compared with *Lepanthes* species previously recorded in Ecuador, including the herbarium collections at QCNE and QCA, as well as in published descriptions and illustrations of the genus from South America, Mexico, and southern Central America.

We assess the extinction risk of the three species following the IUCN (2012) Red List Categories and Criteria. We consider observations, collection sites and individual counts marked during field visits. Species extent of occurrence (EOO) and area of occupancy (AOO) were calculated using GeoCAT (Bachman et al. 2011; http://geocat.kew.org/) with the default 2 km^2^ grid. Based on all available information, we preliminarily evaluate the risk of extinction of each species separately through all the Categories and Criteria.

## Taxonomic treatment

### Lepanthes oro-lojaensis

Taxon classificationPlantaeAsparagalesOrchidaceae

Tobar & M.F.Lopez sp. nov.

7CEB7F38-D3F8-5DED-B731-BD2E73EC1C01

urn:lsid:ipni.org:names:77218876-1

Figs 1 , 2 , 3a

#### Diagnosis.

This species is similar to *Lepanthesjimburae* Luer & Hirtz, but can be distinguished by the smaller plants that are less than 3 cm tall (vs. up to 4 cm tall); the shorter inflorescence that is less than 4 cm long (vs. inflorescence up to 10 cm long), the shorter dorsal sepal with a shorter sepaline tail (6.0 mm vs. 9.0 mm long), the apical lobe of the petals ovate and lower lobe triangular-oblong (vs. petals with subequal, obliquely triangular, acute lobes).

**Figure 1.**
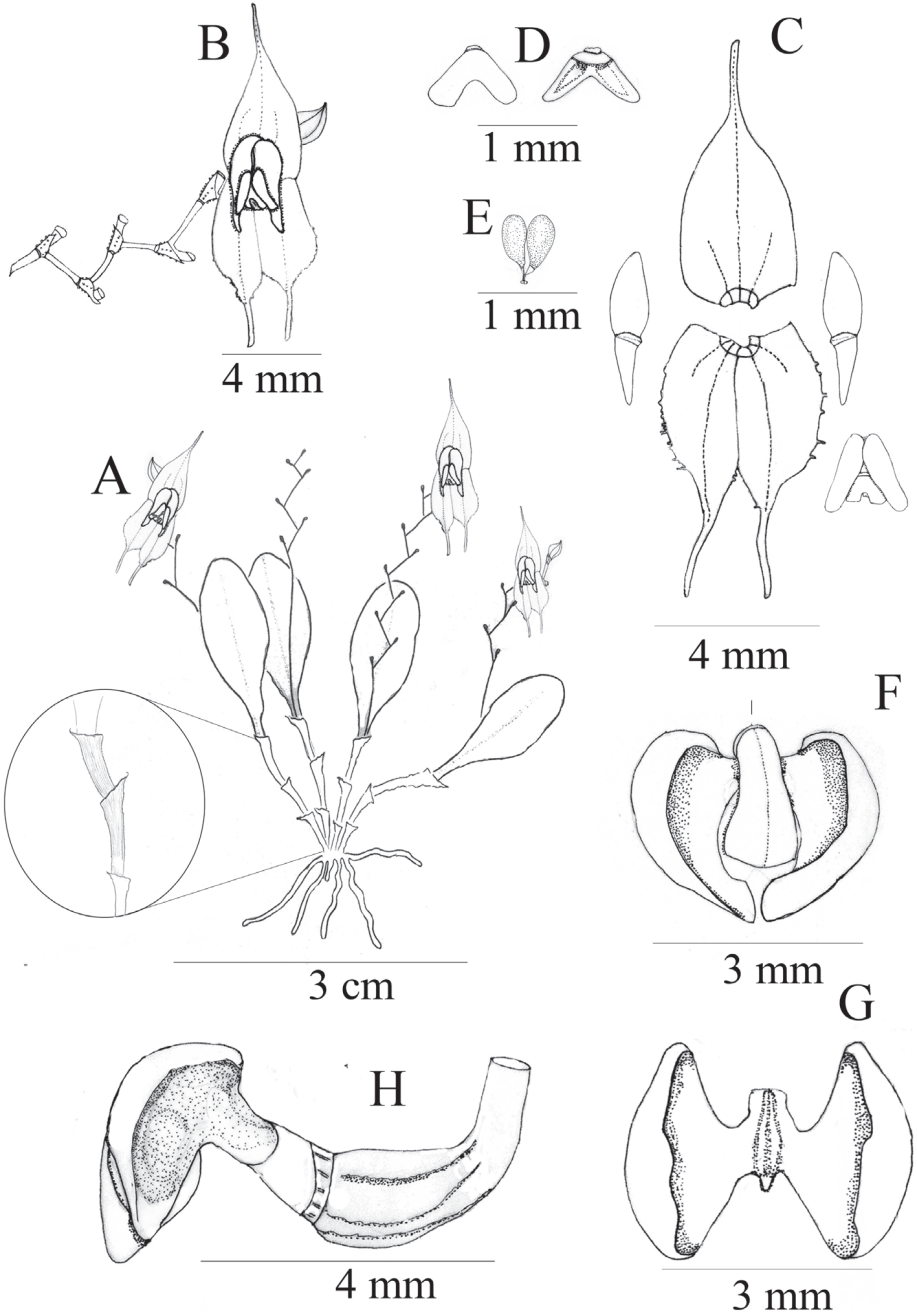
*Lepanthesoro-lojaensis***A** habit **B** flower **C** dissected sepal and petals **D** anther dorsal and ventral view **E** polinarium **F** dorsal view of the spread-out lip with dorsal view of the column **G** dorsal view of the spread-out lip without the column **H** lateral view of the ovary, lip and column. Drawn by F. Tobar & S.Tobar from the plant that served as type (*Tobar et al 1648*).

#### Type.

Ecuador. El Oro, Zaruma, Salvias, near Cerro de Arcos, -3.06963333°N, -79.478944°W, 3500 m, 28 Aug 2015, *Tobar, Gálvez & Obando 1648* (***holotype***: QCNE; ***isotype***: QCA).

**Figure 2.**
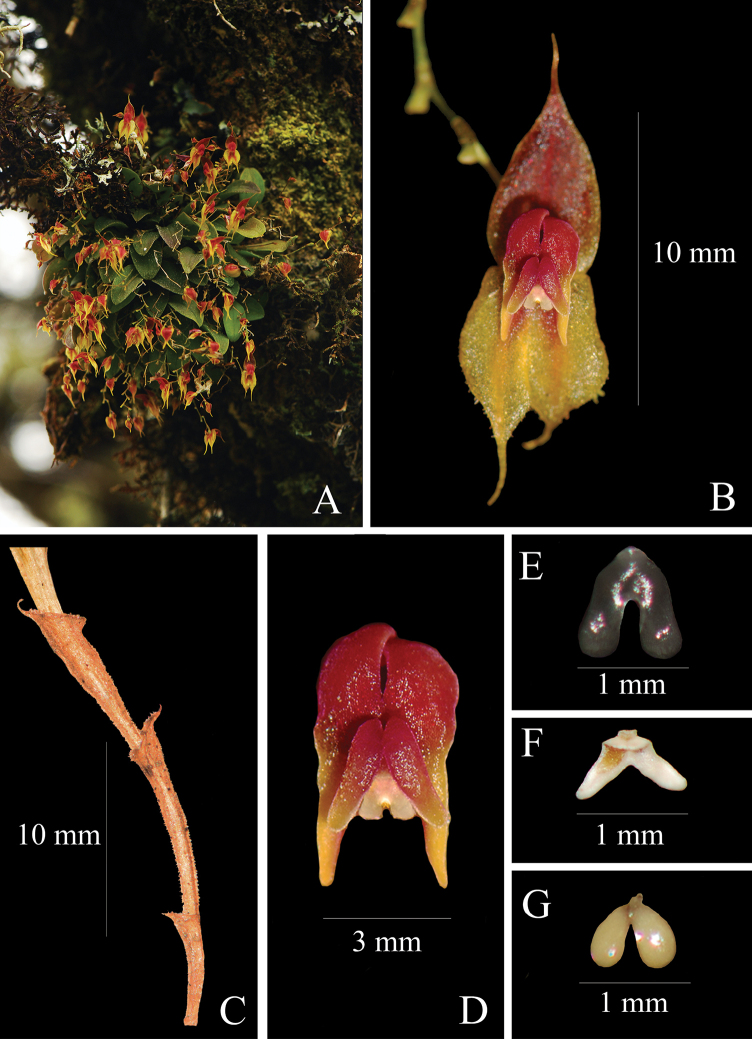
*Lepanthesoro-lojaensis***A** plant growing in its natural habitat **B** front view of the flower **C** detail of the lepanthiform sheat **D** detail of lip and petals **E** anther dorsal view **F** anther ventral view **G** polinarium. Photograph by F. Tobar from the plant that served as type (*Tobar et al. 1648*).

Epiphytic, caespitose herbs up to 3 cm tall. Roots flexuous, cylindrical 0.7 mm in diameter. Rhizome inconspicuous. Ramicaul arcuate to pendulous, 0.9–1.9 × 0.1–0.3 cm long, with 3–4 internodes, covered entirely by light brown minutely puberulent lepanthiform sheaths with a minutely pubescent, acuminate ostium. Leaves dark-green suffused with purple, arcuate 1.0–1.3 × 0.4–0.6 cm, coriaceous, elliptic, subacute to obtuse, tridentate at the apex, base cuneate, contracted into a petiole 2–4 mm long. Inflorescence racemose, one per stem, longer than the leaf, 2.0–3.5 cm long, flexuous, producing 3–16 widely spaced, successively opening flowers; peduncle filiform, 1–3 mm long, surrounded at the base by a bract 1.5 mm long. Floral bracts sub-distichous, infundibuliform, longapiculate. Ovary 1.5 mm long, obpyramidal, slightly arcuate, irregularly keeled. Flowers ca. 12 × 4 mm; dorsal sepal red with a yellow margin, lateral sepals yellow suffused with red around the middle vein; petals with the upper lobe red and the lower one yellow, lip reddish with yellow tips, column reddish, with white and red anther. Dorsal sepal glabrous, slightly concave, ovate, ending in a decurved cauda, 3–veined, 6.0 × 2.7 mm including the cauda. Lateral sepals glabrous, with minutely denticulate margins, connate on their basal one-third, ovate, caudate, 2–veined, 5.0 × 2.0 mm. Petals, bilobate, microscopically pubescent; apical lobe ovate, rounded, lower lobe triangular-oblong, acute, ca. 3.5 × 0.9 mm. Lip bi-laminate, blades ovate, convex, subacute at the base and rounded at the apex, microscopically pubescent, covering most of the column, ca. 1.8 × 0.7 mm; the base of the lip fused to the ventral part of the column, the connectives shortly cuneate, the sinus narrowly oblong with very small, triangular, microscopically pubescent appendix. Column claviform, arcuate, markedly broaden above the middle, truncate at the apex, ca. 1.6 × 0.8 mm. Pollinarium with two ovoid pollinia, with a round, drop-like viscidium. Anther dorsal, deltate. Stigma ventral, horseshoe-shaped. Rostellum minute, apiculate. Capsule globose, 4 × 3 mm.

**Figure 3.**
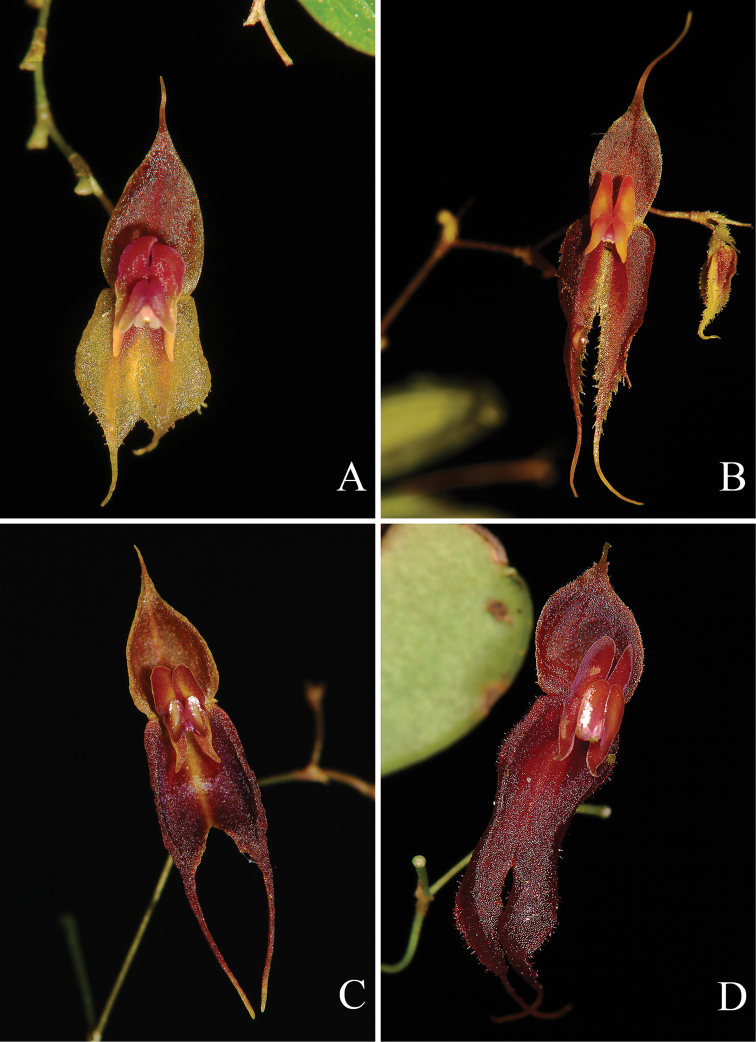
Comparison with the most similar species to *Lepanthesoro-lojaensis* Tobar & M.F.Lopez **A***Lepanthesoro-lojaensis***B***Lepanthesjimburae***C***Lepanthescorkyae***D***Lepanthesschizix*. Photographs by F. Tobar.

#### Distribution and habitat.

**Figure 4.**
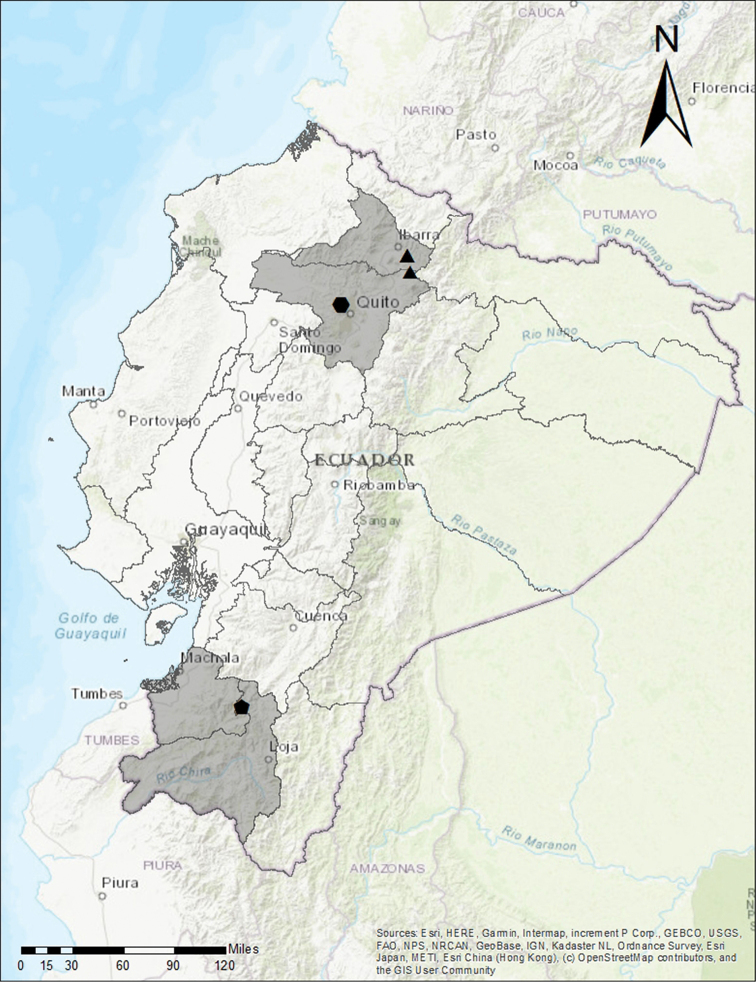
Geographical distribution of the three new species of *Lepanthesmicroprsartima* Tobar & M.J.Gavil. (black hexagon, three collections), *Lepanthescaranqui* Tobar & Monteros (black triangle, two collections) and *Lepanthesoro-lojaensis* Tobar & M.F.Lopez (black pentagon, one collection).

**Figure 5.**
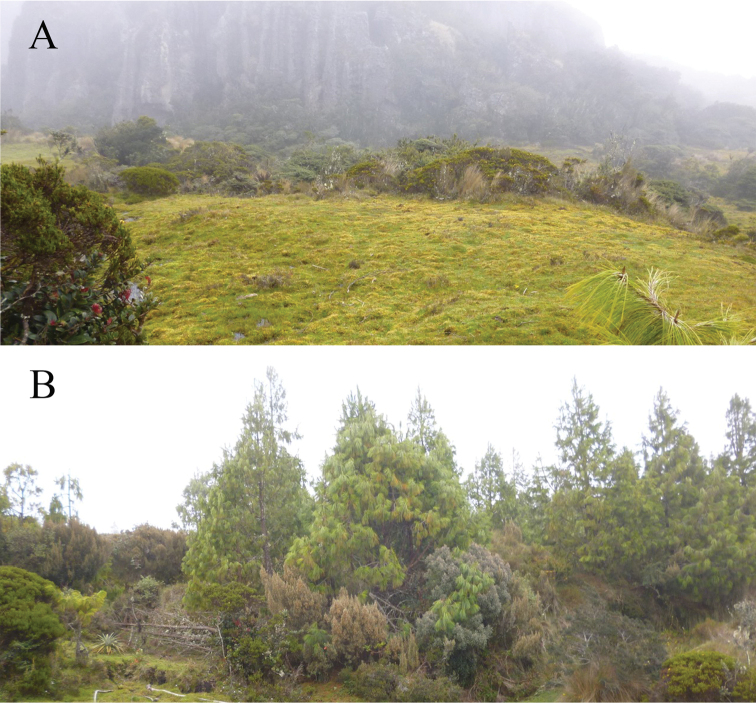
Landscape near to Cerro de Arcos, where *Lepanthesoro-lojaensis* was collected, in El Oro Province **A** natural vegetation remnants **B** areas where *Pinus* have been planted.

*Lepanthesoro-lojaensis* is known from a single locality on the border between El Oro and Loja provinces (Fig. 4). The species was collected in a small patch of scrubs, growing on *Berberislutea* Ruiz & Pav. (Fig. 5), which is a representative species of the evergreen forest formation (BsSn01) according to Ministerio del Ambiente del Ecuador (2013). This type of vegetation is found in sites protected from the wind and desiccation, such as glacial valleys, ravines or under large blocks of rock, which allow them to maintain a higher humidity than the surrounding vegetation and favors the presence of some epiphytes (Sierra et al. 1999).

#### Phenology.

The species was collected in bloom in August and had inflorescences in different stages of development, which suggests that the flowering period may be much broader.

#### Etymology.

The specific epithet refers to the provinces of El Oro and Loja, since this species was discovered at their border.

#### Preliminary conservation status.

*Lepanthesoro-lojaensis*, is known only from the type location, and only two mature individuals were observed. After its discovery in 2015, two additional visits were conducted to explore surrounding areas but it was not possible to find more plants. However, it was evident that the original habitat is under strong pressure due to cattle ranching, the collection of remaining shrubs as firewood and a rapid transformation and fragmentation of the surrounding landscape due to fires and exotic species plantations such as *Pinusradiata* D.Don (Penafiel et al. 2018). Therefore, its small population size, area of occupancy of four km^2^, as well as its habitat restriction and degradation of its unique location allow us to apply criteria B of the IUCN (2012) Red List. The species is preliminarily assessed as Critically Endangered (CR) B2a+b(ii,iii,v) given that it is known from a single location where its area of occupancy, habitat quality, and number of mature individuals are probably declining.

#### Discussion.

Morphologically, the most similar species is *Lepanthesjimburae* (Fig. 3b) from the southeastern slopes of the Ecuadorian Andes. From that species, *L.oro-lojaensis* differs in the smaller plants, the dorsal sepal attenuate into a shorter decurved cauda, the lateral sepals with minutely denticulate margins and petals with the upper lobe ovate, with rounded apex and lower lobe triangular-oblong. The new species is also similar to *L.corkyae* (Fig. 3c) and *L.schizix* (Fig. 3d), both occurring on the western slopes of the northern Ecuador and from which it differs in the red to reddish with yellow dorsal sepals, yellow lateral sepals suffused with red around the mid-vein (vs. orange to red brown sepals in *L.corkyae* and purple flower in *L schizix*), lateral sepals in *L.oro-lojaensis* are minutely denticulate in the margin, are no denticulate in *L.corkyae* and minutely ciliate in *L.schizix*. In Both *L.corkyae* and *L.schizix* the lip blade are glabrous (vs. microscopically pubescent *L.oro-lojaensis*) and, lip blades are oblong in *L.corkyae* and *L.oro-lojaensis* and lunate in *L.schizix*.

### 2. Lepanthes microprosartima

Taxon classificationPlantaeAsparagalesOrchidaceae

Tobar & M.J.Gavil. sp. nov.

037EABB5-DAE7-5EBD-A54C-12A9924F48E7

urn:lsid:ipni.org:names:77218877-1

Figs 6 , 7 , 8a

#### Diagnosis.

Similar in habit to *Lepanthesobandoi* Tobar & M.F. López, but distinguished by the inflorescence shorter than the leaf (vs. Inflorescence longer than) and petals with unequal triangular lobes (vs. lobes lanceolate-oblong, subequal). *Lepanthesmirador* Luer & Hirtz is also similar, differing from it in the superposed, arcuate secondary stems (vs. secondary stems erect, not superposed), leaf light green on the underside (vs. dark purple underside), and the tiny, oblong-lanceolate appendix (vs. appendix oblong with bilobed apex).

**Figure 6.**
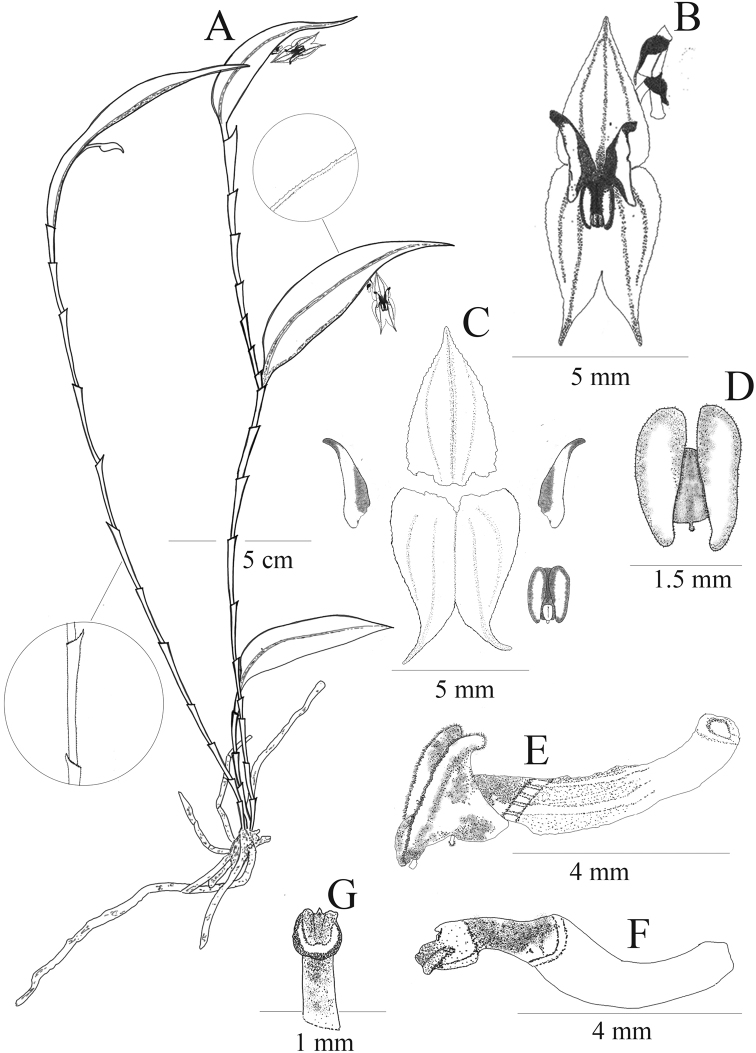
*Lepanthesmicroprosartima***A** habit **B** flower **C** dissected sepal and petals **D** dorsal view of the spread-out lip whitout the column **E** lateral view of the ovary, and lip **F** lateral view of the ovary and column **G** ventral view of the column showing the horse-shaped stigma Drawn by M. Gavilanes from the plant that served as type (*Tobar et al. 3357*).

#### Type.

Ecuador. Pichincha, Nono, Yanacocha Reserve, masked trogon path, 0.122416°N, -78.590283°W, 3530 m, 25 Nov 2018, *Tobar & Angulo 3357* (***holotype***: QCA-spirit; ***isotypes***: QCNE, HPUCESI-spirit).

Terrestrial, caespitose, prolific herbs up to 40 cm in height. Roots flexuous, cylindrical, pink with yellow apex. Ramicauls arcuate, new stems arise from the apex of the old ones superposed, 4.1–25.0 × 0.2–0.3 cm long, with 4–16 internodes, covered completely by lepanthiform sheaths, these light brown, 0.3–2.9 cm long, the ostium microscopically muricate, acuminate. Leaves arcuate, 7.5–9.4 × 1.1–2.2 cm, blades oblong-ovate, light to dark green, minutely serrate along the margin, long-acuminate apically, base cuneate, contracted into a petiole 4–7 mm long. Inflorescence one per stem, shorter than the leaf, 2.5–6 cm long, borne on the underside of the leaf, racemose; peduncle filiform, 2 mm long, ca. 0.5 mm in diameter, surrounded by a basal bract. Floral bracts 2 mm long, papiraceous, obliquely infundibuliform, glabrous and long-apiculate. Ovary 3.2 mm long, obpyramidal, with irregular keels. Flowers ca. 4.5 × 13 mm; sepals entirely yellow, petals yellow with edges slightly suffused with red or pink; lip yellow with the base and edges of the blades red or pink; column pink or purple and anther purple with two yellow spots at the base. Sepals with minutely denticulate margins, dorsal sepal 6 × 4.5 mm, broadly ovate-triangular, minutely denticulate, shortly acuminate, 3-veined; lateral sepals 7 × 2.4 mm long, connate to their middle, obliquely ovate with divergent acute-acuminate apex, 2-veined. Petals ca. 3 × 1.4 mm long, 1-veined, minutely pubescent, transversely bilobed, the upper lobe narrowly triangular with revolute margins, the lower lobe smaller, broadly triangular, obtuse. Lip bi-laminate, the blades minutely pubescent, ovate, rounded, close to each other in their proximal part and divergent at the apex, ca. 1.4 ×1.2 mm; connective short, deeply cuneate, the base of the lip connate with the base of the column, sinuous, obtuse; appendix tiny, oblong-lanceolate, pubescent at the apex. Column slightly arcuate, slightly broadened apically, somewhat compressed dorsoventrally, ca. 1.2 × 0.8 mm; clinandrium covering the lower half of the anther. Anther dorsal, stigma ventral, horseshoe-shaped. Rostellum minutely triangular, yellow. Capsule ovoid 6-ribed ca. 4 × 6 mm, with persistent perianth. Capsule ellipsoid, 6-ribed.

**Figure 7.**
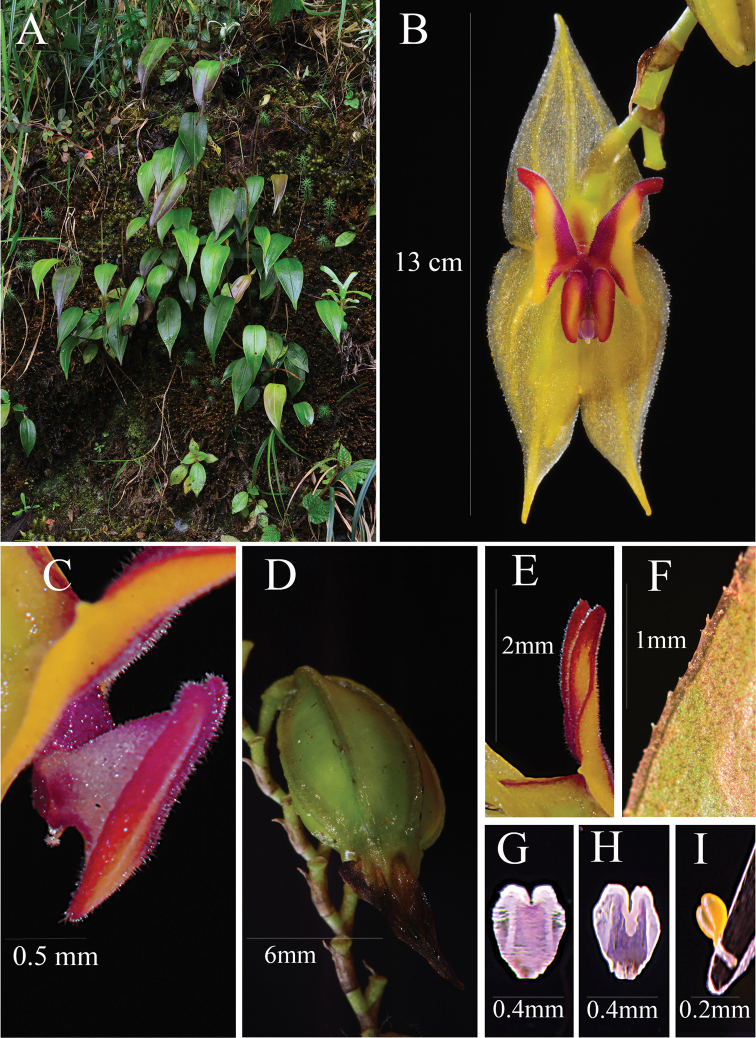
*Lepanthesmicroprosartima***A** plant growing in its natural habitat **B** front view of the flower **C** lateral view of the lip showing the apêndix **D** detail of the fruit **E** lateral view of the petal showing the revolute apex **F** detail of the leaf margin, minutely denticulate **G** anther dorsal view **H** anther ventral view **I** polinarium. Photograph by Francisco Tobar from the plant that served as type (*Tobar et al. 3357*).

#### Other specimens examined.

***Paratypes*** Ecuador. Pichincha, Nono, Reserva Verdecocha, Verdecocha: Transecto de Aves y Conservación en Reserva Verdecocha, -0.118420°N, -78.597470°W, 3400 m, 06 Feb 2018, *Tobar, Santander & Hipo 3130* (QCA); Nono, Yanacocha Reserve, sendero hacia la Reserva Verdecocha 500 metros al suroeste de los bebederos de colibríes, 0.118420°N, -78.597470°W, 3810 m, 07 May 2018, *Tobar 3359* (QCA).

#### Distribution and habitat.

This species is endemic to the Yanacocha and Verdecocha reserves on the western slopes of Volcán Pichincha (Fig. 4), where it is found growing from 3200 to 3800 m in evergreen montane forest (BsAn03) according to Ministerio del Ambiente del Ecuador (2013). *Lepanthesmicroprosartima* grows terrestrially on the edges of the trails of both reserves and shares the habitat with other species such as *L.mucronata* Lindl., *L.bibalbis* Luer & Sijm and *L.dunstervilleorum* Foldats, as well as *Stelislaevigata* (Lindl.) Pridgeon & M.W.Chase, *Stelispusilla* Kunth, *Masdevallialaevis* Lindl. and *Platystelestonyx* Luer. Unlike other terrestrial species of *Lepanthes* that grow on roadside embankments with greater availability of light, this species can also thrive within the forest in dense shade.

#### Phenology.

The species was collected in flower in November, February and May, which suggests that flowering occurs throughout the rainy season, from October to the end of May.

#### Etymology.

From the Greek μικρό, small and προσάρτημα, appendix, in reference to the tiny appendix of this species.

#### Preliminary conservation status.

Only three collecting sites have been found during three years of monitoring at two locations: Yanacocha and Verdecocha reserves (Fig. 9), and around 40 mature individuals are known, which suggests that it is a rare species. This orchid is mainly terrestrial, and has not been found growing in other trails of the reserve or in nearby areas, the extent of occurrence calculate for the specie is < 100 km^2^ and area of occupancy is 8 km^2^, Based on the available information, this species is preliminarily assessed as Critically Endangered (CR) B1a+2a given that the known population are restricted to a small area in the western slopes of Pichincha Volcano, representing one location (sensu IUCN 2012), and the number of known mature individuals is fewer than 250.

#### Discussion.

**Figure 8.**
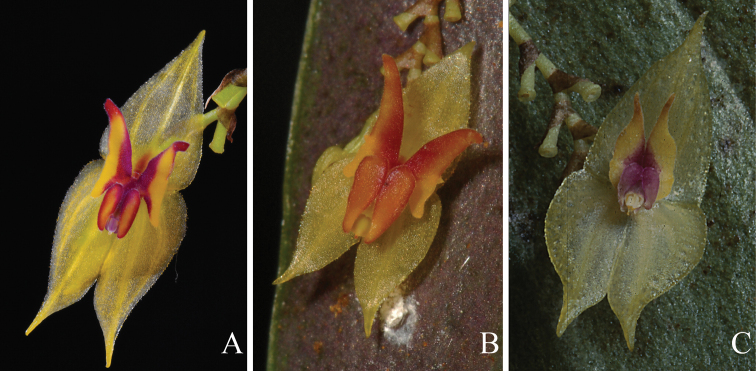
Comparison with the most similar species to *Lepanthesmicroprosartima* Tobar & M.J.Gavil. **A***Lepanthesmicroprosartima***B***Lepanthesmirador***C***Lepanthestungurahuae*. Photographs **A, B** by F. Tobar and **C** A. Hirtz.

The closest species are *Lepanthesmirador* (Fig. 8a) from north-east Ecuador and Central Cordillera of Colombia, and *L.tungurahuae* Luer & Hirtz (Fig. 8c) from central Ecuador, but is easily distinguished from both by the overlapping secondary stems. *Lepanthesmicroprosartima* also differs from *L.mirador* in the light green leaves and oblong-lanceolate appendix (vs. leaves dark purple in the abaxial surface and appendix oblong, with a bilobed apical segment). From *L.tungurahuae* the new species is distinguished by the oblong leaves (vs. ovate elliptical), the petal lobes revolute, marked with red at the edges (vs. not revolute and marked with red at the base) and the blades of the labellum ovate (vs. blades narrowing oblong-ovate). In habit it also resembles *L.obandoi* from the north east of Ecuador, but the new species has an inflorescence shorter than the leaf (vs. longer than the leaf.), and the petals have triangular, unequal lobes (vs. lobes subequal, lanceolate-oblong).

**Figure 9.**
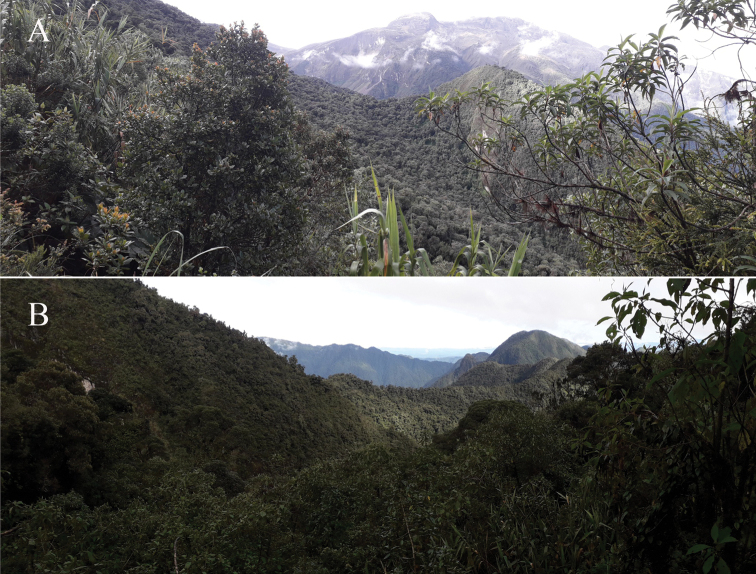
Natural habitat of *Lepanthesmicroprosartima* in the western slopes of Pichincha volcano **A** Yanacocha reserve **B** Verdecocha reserve.

### 3. Lepanthes caranqui

Taxon classificationPlantaeAsparagalesOrchidaceae

Tobar & Monteros sp. nov.

4F788887-B990-5D22-86BE-6707B5992B2E

urn:lsid:ipni.org:names:77218878-1

Figs 10 , 11 , 12a

#### Diagnosis.

Similar to *Lepanthespachychila* Luer & Hirtz, differing in the taller plants up to 40 cm long (vs. less than 20 cm tall), the petals with narrowly triangular-oblong lobes (vs. lobes triangular), the lip with the blades thin, ovate-oblong, the base rounded and apically acute (vs. lip blade thick, broadly ovate with basal and apically rounded ends) and appendix triangular in dorsal view, with two protuberances on the top and a minute tuft of hairs at the base (vs. minutely bilobulate appendix).

#### Type.

Ecuador. Pichincha, Cayambe, Olmedo, El Chalpar, 5 km northwest of the San Marcos Lagoon, 3500 m, 00.15211°N, -78.00220°W, 20 Jul 2019, *Tobar, Jaramillo, Correa & Monteros 3348* (***holotype*** QCA, spirit; ***isotypes*** QCNE, HPUSECI).

Terrestrial, caespitose, prolific herbs up to 40 cm in height. Roots flexuous, cylindrical, deep pink. Ramicauls arcuate or pendulous, with 6–12 internodes, 4–22 × 0.2–0.8 cm long, covered completely by lepanthiform sheaths, these light brown, papillose, 0.5–2.5 cm long, the ostium microscopically muricate, acuminate. Leaves arcuate, slightly concave, 3.5–9.0 × 0.8–2.4 cm, blades ovate to oblong, light to dark green, long-attenuate, tridenticulate apically, base cuneate, contracted into a petiole 1–3 mm long. Inflorescence 1.0–5.6 cm long, shorter than the leaves, racemose, densely flowered, one or six per stem, producing one or two successively opening flowers; peduncle filiform, 1.0–1.5 mm long, surrounded by a basal bract. Floral bracts 2 mm long, distichous, glabrous, apiculate. Ovary 3 mm long, obpyramidal, with 6 irregular keels. Flowers ca. 13 × 8 mm; sepals minutely denticulate, entirely light yellow; petals pubescent, yellow with proximal part of the upper lobe red to brown, lip minutely pubescent white with yellow, with the base and edges of the blades purple or brown, column pink and yellow, anther white with purple apex. Dorsal sepal 7.0 × 5.0 mm, broadly ovate, shortly acuminate, 3-veined. Lateral sepals 2-veined, 6.0 × 4.0 mm, connate at least on their proximal two-thirds, obliquely ovate with divergent, shortly acuminate apices. Petals 1-veined, ca. 4.5 × 1.5 mm, transversely bilobed, lobes subequal, narrowly triangular-oblong, rounded. Lip with blades ovate-oblong, microscopically pubescent, close to each other in their proximal part and divergent at their apices, not covering the column, base of the blades rounded, apical part acute, incurved, ciliate, ca. 2.0 × 1.6 mm; connective broadly cuneate, minutely pubescent, its body connate with the base of the column, sinus obtuse, with a small, rounded, pubescent appendix, which has two protuberances on the top and a minute tuft of hairs. Column claviform, straight, ca. 2.0 × 0.8 mm; clinandrium covering only the lower half of the anther. Anther dorsal, stigma ventral. Rostellum more or less oblong with the apex rounded, yellow. Capsule not seen.

**Figure 10.**
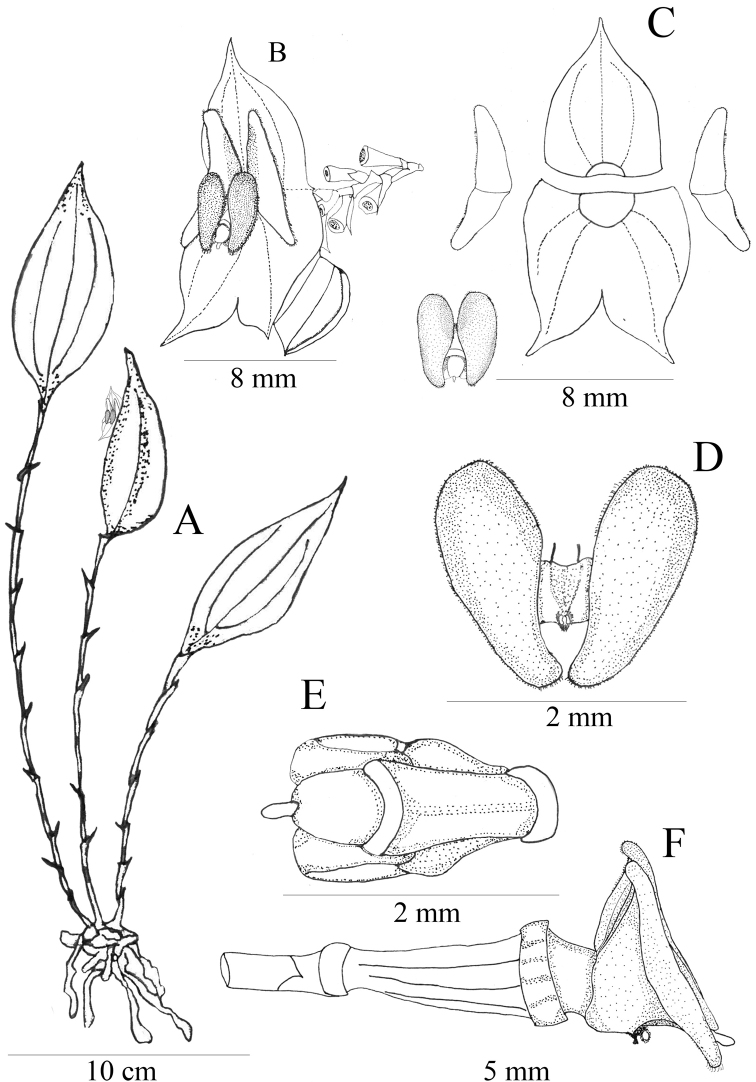
*Lepanthescaranqui***A** habit **B** flower **C** dissected sepal and petals **D** dorsal view of the spread-out lip without the column **E** dorsal view of the column **F** lateral view of the ovary, and lip. Drawn by F. Tobar from the plant that served as type (*Tobar et al. 3348*).

#### Other specimens examined.

***Paratypes*** Ecuador. Imbabura, Ibarra, El Sagrario, forest near La Carbonería, 3732 m, 0.310255°N, -78.066891°W, 15 May 2017, *Tobar, Monge & Obando 2498* (HPUCESI, spirit).

**Figure 11.**
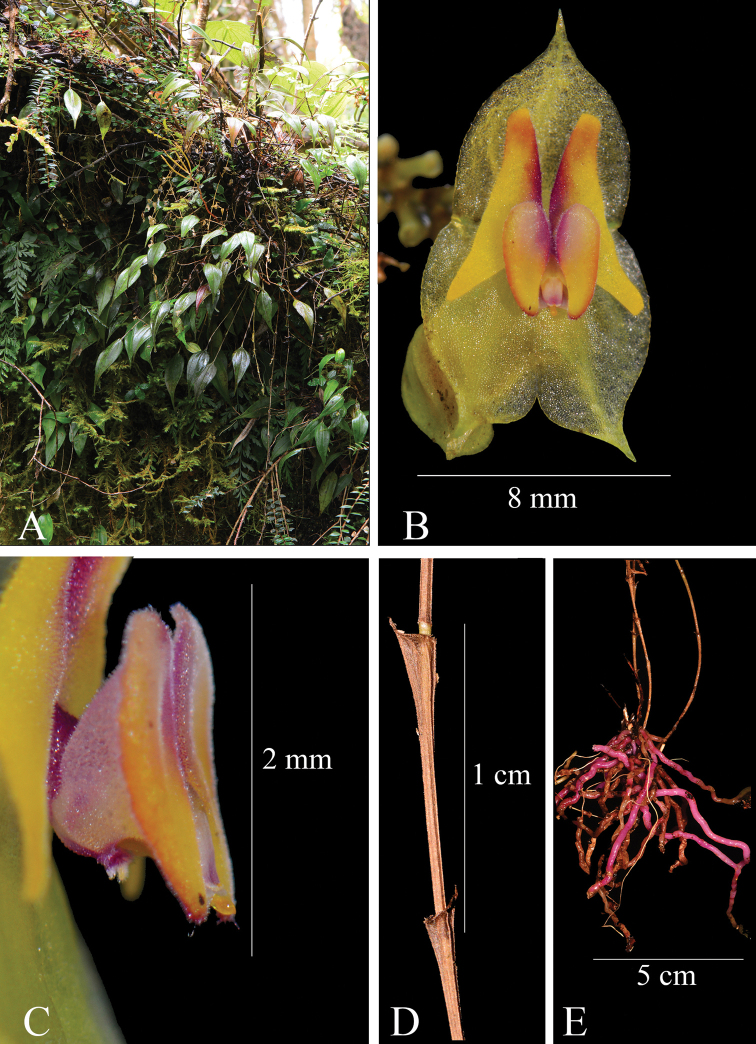
*Lepanthescaranqui***A** plant growing in its natural habitat **B** front view of the flower **C** lateral view of the lip showing the apêndix **D** detail of the lepanthiform sheat **E** roots detail. Photograph by F. Tobar from the plant that served as type (*Tobar et al. 3348*).

#### Distribution and ecology.

This species was collected in the buffer zone of the Cayambe-Coca National Park on the eastern Imbabura and Pichincha provinces (Fig. 4). The population from Imbabura (Fig. 13) grows in páramo (AsSn01) according to Ministerio del Ambiente del Ecuador (2013) as small groups or isolated individuals that grow on roadside embankments along with other members of Pleurothallidinae like *Draconanthesaberrans* (Schltr.) Luer, *Stelispusilla*, *S.lamellata* Lindl., *Pleurothallisbivalvis* Lindl. and *P.apopsis* Luer. The specimens collected in Pichincha grew in evergreen montane forest (BsAn01)(Fig. 13) according to Ministerio del Ambiente del Ecuador (2013), and unlike the Imbabura population, the plants grow epiphytically at the base of the trunks or on the lower branches of the trees, where they also share their habitat with *Stelispusilla*, *S.lamellata*, *Pleurothallisbivalvis* and *P.apopsis*.

#### Phenology.

The species has been found in flowers and with fruits at different stages of maturity from May to July, suggesting that reproduction takes place all year round.

#### Etymology.

The specific epithet honors the Caranqui culture that historically occupied the same areas where this species is distributed.

#### Preliminary conservation status.

*Lepanthescaranqui* is known from two localities within an extent of occurrence of 575 km^2^. It inhabits both paramo and montane forest where it is more abundant, forming small colonies on tree trunks. Its habitat is not considered to be under pressure since it is located in the buffer zone of a protected area but a potential threat would be the advance of the agricultural frontier. However, it has been observed that this orchid can adapt to moderately disturbed areas and is able to colonize different types of vegetation. Considering the abundant number of mature individuals observed in the field we estimate an approximate number of 500 mature individual and giving that its area of occupancy, habitat quality and the number of mature individuals are not declining we suggest the Least Concern (LC) category following the IUCN (2012) Red List Categories and Criteria.

#### Discussion.

**Figure 12.**
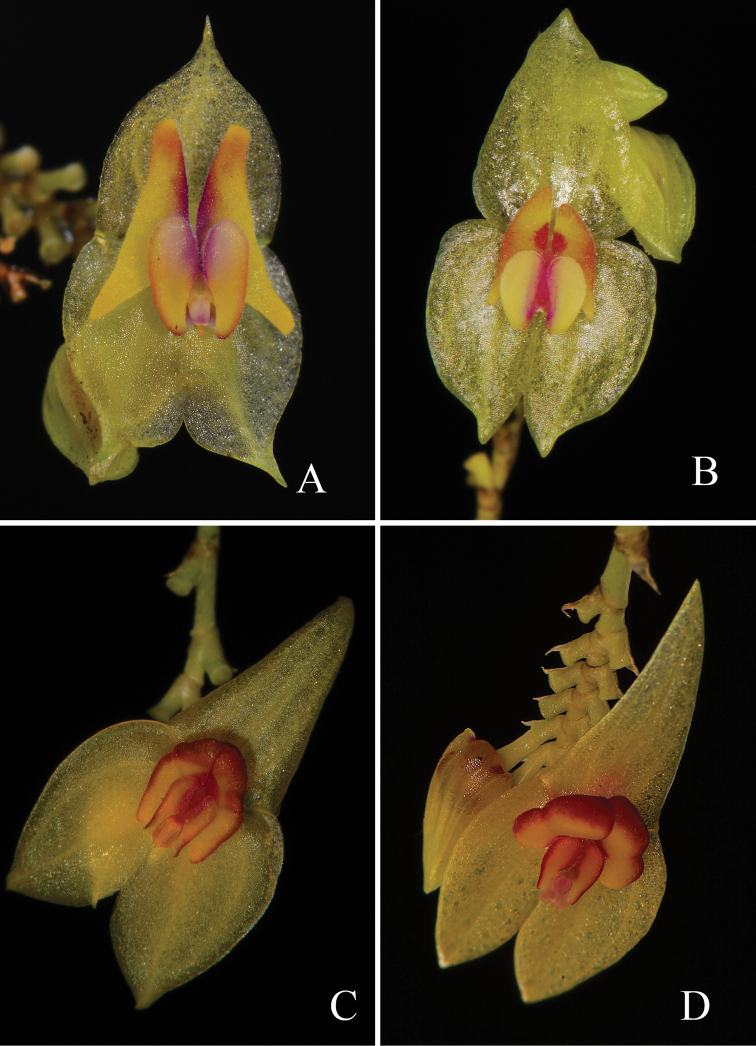
Comparison with the most similar species to *Lepanthescaranqui* Tobar & Monteros **A***Lepanthescaranqui***B***Lepanthespachychila***C***Lepantheschrysina***D***Lepanthesballatrix*. Photographs by F. Tobar.

*Lepanthescaranqui* is morphologically most similar to *L.pachychila* (Fig. 12b) from the southwest of Ecuador, it differs in having taller plants, petals narrowly triangular-oblong, the lip blade thick, broadly ovate with rounded ends, appendix triangular in the dorsal view, with two protuberances on the top and a minute tuft of hairs at the base. The new species also resembles *L.ballatrix* (Fig. 12c) which is widespread in Ecuador and Colombia and *L.chrysina* (Fig. 12d) endemic from the southwest of Ecuador. Both species have a triangular, acute dorsal sepal (vs. broadly-ovate, narrowly acuminate). The petal lobes in *L.ballatrix* are suborbicular to broadly elliptical, and in *L.chrysina* the upper lobe is oblong, obtuse and the lower obliquely triangular (vs. petals equal, narrowly triangular-oblong in *L.caranqui*), the lip blades is glabrous in *L.chrysina* and minutely pubescent in *L.ballatrix* and *L.caranqui*, and are oblong lunate in *L.ballatrix* and ovate-oblong in *L.chrysina* and *L.caranqui*. The appendix in *L.caranqui* is triangular pubescent with two protuberances on the top, and in *L.ballatrix* is triangular, minutely pubescent, thickened at the end, with a pair of minute finger like process.

**Figure 13.**
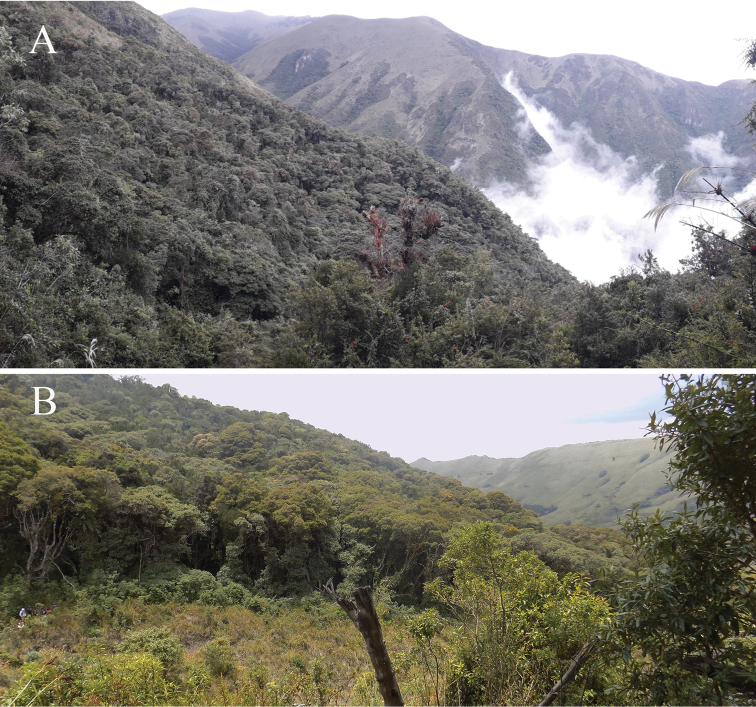
Natural habitat of *Lepanthescaranqui***A** Paramo of La Carboneria in east of Imbabura province **B** El Chalpar Area east of Pichincha province. Photographs by F. Tobar and M. Monteros.

## Supplementary Material

XML Treatment for Lepanthes oro-lojaensis

XML Treatment for Lepanthes microprosartima

XML Treatment for Lepanthes caranqui
